# Subversion of host cell signaling: The arsenal of Rickettsial species

**DOI:** 10.3389/fcimb.2022.995933

**Published:** 2022-10-31

**Authors:** Dan Huang, Jingjing Luo, Xuan OuYang, Lei Song

**Affiliations:** ^1^ Department of Respiratory Medicine, Center of Pathogen Biology and Infectious Disease, Key Laboratory of Organ Regeneration and Transplantation of the Ministry of Education, The First Hospital of Jilin University, Changchun, China; ^2^ State Key Laboratory of Pathogen and Biosecurity, Beijing Institute of Microbiology and Epidemiology, Beijing, China

**Keywords:** Type IV secretion system, effector, virulence, invasion, actin, phosphoinositide

## Abstract

Rickettsia is a genus of nonmotile, Gram-negative, non-spore-forming, highly pleomorphic bacteria that cause severe epidemic rickettsioses. The spotted fever group and typhi group are major members of the genus Rickettsia. Rickettsial species from the two groups subvert diverse host cellular processes, including membrane dynamics, actin cytoskeleton dynamics, phosphoinositide metabolism, intracellular trafficking, and immune defense, to promote their host colonization and intercellular transmission through secreted effectors (virulence factors). However, lineage-specific rickettsiae have exploited divergent strategies to accomplish such challenging tasks and these elaborated strategies focus on distinct host cell processes. In the present review, we summarized current understandings of how different rickettsial species employ their effectors’ arsenal to affect host cellular processes in order to promote their own replication or to avoid destruction.

## Introduction

As Gram-negative and obligate intracellular micro-organisms, rickettsiae belong to the order Rickettsiales, Alphaproteobacteria (Walker and Ismail, 2008; Chan et al., 2010). Blood-feeding arthropods are intermediate vectors, and eventually, rickettsial pathogens are transmitted to humans by arthropods, resulting in mild to life-threatening diseases with symptoms as follows: fever, rash, headache, splenomegaly, and lymphadenopathy. Rickettsial infections have a long history, dating from antiquity, and have had a great influence on the history and evolution of humanity (Quintal, 1996). With the development of medicine and science, the definition and classification of rickettsial organisms are more reasoned and accurate when combined with clinical manifestation and molecular methods. These include the methods described in Bergey’s Manual of Systematic Bacteriology, 16S rRNA gene sequencing (Fournier and Raoult, 2009), and whole genome sequencing (Leclerque, 2008). Species within the genus Rickettsia can be categorized into four groups as follows: ancestral group (AG), including *R*. *canadensis* and *R*. *bellii*, which are the most distantly related rickettsiae; typhi group (TG), including *R*. *typhi*, which is the causative agent of endemic typhus dependent on rat fleas as a vector, and *R*. *prowazekii*, which is the etiologic agent of epidemic typhus dependent on louse dissemination to humans; transitional group (TRG), including *R*. *australis*, which is the agent responsible for Queensland tick typhus, *R*. *felis*; and spotted fever group (SFG), such as *R*. *rickettsii*, the causing agent of Rocky Mountain spotted fever (RMSF), in America and *R*. *conorii*, the causing agent of Mediterranean spotted fever (MSF) in Europe (Gillespie et al., 2009a).

The obligate intracellular survival and proliferation of rickettsial species are significantly different from those of facultative intracellular bacteria such as *Legionella pneumophila*, *Listeria monocytogenes*, and *Salmonella Typhimurium*. The reductive evolution of rickettsial genomes leads to succinct genomes that are dependent on the host cell substrate for a wide range of biochemical reactions. Because the survival of rickettsial species without host cells is not possible with reductive genomes (Wood and Azad, 2000), they have evolved sophisticated mechanisms to export various virulence factors to survive in host cells. For example, some of the effectors are secreted on the surface of *rickettsia* spp. in order to adhere to and invade host cells. The others are translocated into nutrient-rich cytoplasm, subverting host signaling pathways, evading innate immune defense, and developing a beneficial replication niche (Martinez and Cossart, 2004).

The transmission of *rickettsia* spp. to humans is *via* the bite of blood-sucking vectors or inhalation of arthropod feces. *Rickettsia* spp. infect microvascular endothelial cells and immune cells, such as macrophages and monocytes, in the initiation stage. When multiple bacteria lyse host cells and spread *via* the lymphatic system, a series of clinical manifestations occurs (Sahni et al., 2019). *Rickettsia* spp. increases its infective efficiency by activating phagocytosis in non-phagocytic cells through secreted adhesins and the interaction with the host cell receptors, which greatly contribute to successful entry into the cytoplasm by stimulating cytoplasmic signaling cascades (Martinez and Cossart, 2004). Escape from the phagosome or endosome is another step for *rickettsia* spp. to overcome and translocated virulence factors play predominant roles in this process, exemplified by phospholipases (PLs) (Rahman et al., 2013) and PI (phosphoinositide) kinases (Voss et al., 2020). The polymerization of cytoskeletal actin and the rearrangement of the PM (plasma membrane) are demonstrated to be significant for bacterial invasion and transmission, which are hijacked by *rickettsia* spp. (Teysseire et al., 1992; Chan et al., 2010). Given the replicated lifestyle of intracellular bacteria, the manipulation of the innate immune signaling pathways, such as autophagy (Huang and Brumell, 2014; Xu et al., 2019; Fu et al., 2022c), nuclear factor-kappaB (NF-κB) signaling pathway (Radulovic et al., 2002; Zhang et al., 2022; Fu et al., 2022b), and caspase-11-GSDMD-IL-1α signaling axis (Voss et al., 2022), is also an essential step for bacterial infection. Multiple virulence factors are involved in these biological functions, some of which are highly conserved, and others are specific or variable across species, indicating that species-specific analysis is necessary. Besides, unlike the functional redundancy in the effectors of *Legionella* species (Song et al., 2021; Song et al., 2022; Fu et al., 2022a), each effector is crucial for the adherence, invasion, and intracellular replication of rickettsiae. Hence, we reviewed multiple rickettsial effectors implicated in the invasion, escape, replication, and spreading of unique species of the genus Rickettsia and elucidated new insights on how these virulence factors attenuate the host’s innate immune defense and orchestrate the permissive replication niche.

## Effectors from *Rickettsia* spp. of SFG

### Effectors involved in bacterial adherence and invasion

*R*. *rickettsii* and *R*. *conorii* are etiologic agents of RMSF and MSF, respectively. They are the most virulent infections spread to humans by infected ticks among all rickettsioses with the potential of fatality, especially in young people (Quintero Velez et al., 2019). Upon the tick bite, the bacterium is transmitted into a small-to-medium-sized blood vessel, leading to increased vesicular permeability and fluid leakage and eventually causing continuous skin and tissue damage, such as rash, skin necrosis, and gangrene (Muzumdar et al., 2019). *Rickettsia* spp. are highly evolutive organisms that deploy multiple surface proteins (Gong et al., 2014) for invading the vascular endothelial cells and establishing a replication niche within a nutrient-rich cytoplasm.

The surface proteins of bacteria indicate that they are in the first line of interaction with eukaryotic cell components. Extensive experimental evidence suggests that members of the Sca (surface cell antigen) family mediate interactions with host cell membranes during the initial phases of infection. The family of Sca proteins, namely Sca0 (OmpA), Sca1-4, Sca5 (OmpB), and other putative Sca 6-16 proteins (Blanc et al., 2005) are conserved and specific across *rickettsia* spp. Nevertheless, Sca1, Sca4, and Sca5 (OmpB) are highly conserved amongst all *rickettsia* spp.

Most Sca proteins, except Sca4, belong to the family of autotransporters (ATs) (also namely sec-dependent T5SS: type V secretion system), which have three modular domains, including an N-terminal (NT) signal sequence (SS), a central passenger domain, and a C-terminal translocation module (β-peptide) (Chan et al., 2010; Gillespie et al., 2015). The Sec translocon anchors in the bacterial inner membrane (IM) and is composed of the IM and cytosolic sections that act as a pathway to interact with Sca proteins through the NT helical transmembrane spanning (TMS) region and/or a strongly hydrophobic SS. The Sca proteins passage the Sec channel, then are secreted into the periplasm (PP) and complete the process from the IM to the PP. The NT SS is recognized and cleaved by signal peptidase (SPase) I. As a consequence, the C-terminal domain inserts into the outer membrane to form a β-barrel-rich transmembrane pore, which is responsible for transporting the passenger domain into the extracellular matrix (Gillespie et al., 2015). Collectively, the Sca proteins, the subset of rickettsial secreted proteins, are sec-dependent ATs localized on the surface of *R*. *rickettsii*, which contribute greatly to adherence and invasion into host cells.

*R*. *rickettsii* possesses two major surface-exposed proteins of outer membrane protein A (OmpA molecular mass: 190 kDa) and outer membrane protein B (OmpB molecular mass: 120 kDa) (Dantas-Torres, 2007; Noriea et al., 2017). OmpB, also termed Sca5, is the most abundant protein in the outer membrane of *R*. *rickettsii*. The ectopic expression of the gene, OmpB, from the *R*. *japonica* genome in *E*. *coli* BL21 (DE3) is sufficient for bacterial adherence and invasion into Vero cells (Uchiyama et al., 2006). OmpB is the ligand of cell surface protein Ku70, which is a subunit of DNA-dependent protein kinase (DNA-PK), a serine/threonine kinase homologous to the PI3-kinase superfamily (Martinez et al., 2005). The interaction between OmpB and Ku70 is critical for the infection of non-phagocytic mammalian cells. The ubiquitin ligase of the host cell, c-Cbl, mediates the ubiquitination of Ku70 and contributes to *R*. *conorii* entry. The suppression of the endogenous expression of c-Cbl at the protein level reduces the *R. conorii*-induced ubiquitination of Ku70, which alleviates the invasion of *R*. *conorii* (Chan et al., 2009). Professor Martinez and his colleagues have further illustrated that OmpB-mediated infection culminates in actin recruitment at the bacterial foci, and actin polymerization is required in this entry process. A series of experimental results have also verified that clathrin and caveolin-dependent endocytic events likely contribute to the internalization process (Chan et al., 2009).

OmpA, also termed Sca0, mediates the adherence and invasion of *R*. *conorii*. The heterodimeric protein, α2β1 integrin, is a specific mammalian internalization receptor of OmpA, and blocking or decreasing the expression of α2β1 integrin markedly diminishes *R*. *conorii* invasion of endothelial cells (Hillman et al., 2013). Truncated OmpA and defects in the processing of OmpB are reasons why OmpA and OmpB are absent on the surface of avirulent strains (Ellison et al., 2008; Noriea et al., 2017). However, a single knockout of OmpA in the highly virulent Sheila Smith strain does not cause attenuation in a guinea pig model of the infection and entry into the endothelial cells (Noriea et al., 2015). These data further emphasize the critical role of OmpA and OmpB in bacterial entry into host cells.

Sca1 is highly conserved among SFG rickettsial species, including *R*. *africae*, *R*. *rickettsii* Sheila Smith, and *R*. *japonica* (Ngwamidiba et al., 2006). Ectopically expressed Sca1 in the *E*. *coli* system demonstrates that Sca1 is involved in *R*. *conorii* adherence to the host cells but not in the process of invasion (Riley et al., 2010). The presence of Sca2 in the *R*. *conorii* genome and the genome of virtually all SFG rickettsial species (Ngwamidiba et al., 2006; Cardwell and Martinez, 2009) suggests that Sca2 plays a critical role during the life cycle of rickettsiae. The expression of Sca2 at the outer membrane of *E*. *coli* is sufficient to mediate adherence to, and invasion of, various mammalian cells even in the absence of other rickettsial virulence factors. Furthermore, scanning and transmission electron micrographs show that the pretreatment of Vero cells with soluble Sca2 protein can reduce the invasion of *R*. *conorii* during infection (Cardwell and Martinez, 2009).

Besides, proteomic analysis using rickettsial species has identified that Sca family proteins contribute to bacterial entry into host cells, and there are two adhesions: Adr1 and Adr2 (Renesto et al., 2006; Gong et al., 2014). Adr1 and Adr2 are highly conserved across all rickettsial species, and their coding genes are *RC1281* and *RC1282* in the *R*. *conorii* genome, respectively. Sequestering by an anti-Adr2 monoclonal antibody reduces bacterial entry and rickettsiae-induced cytotoxicity (Vellaiswamy et al., 2011). However, the function of Adr1 is different from Adr2, which inhibits the complement system in order to help bacteria evade killing by innate and adaptive immune systems (Riley et al., 2014). It is well known that complement systems are responsible for killing and clearance of bacterial pathogens *via* large pore-forming complexes or the recruitment of immune cells (Heesterbeek et al., 2018). Although different bacteria interplay with different complement system regulators (Singh et al., 2010; Hallstrom et al., 2015; Syed and Wooten, 2021), the purpose is always to escape from complement-mediated killing. It is identified that a series of regulatory proteins, including factor I, factor H, C4-binding protein, vitronectin, and clusterin, regulate and block complement system activation. Adr1 of *R*. *conorii*, through double lysine amino acid residues located within loops 3 and 4 bound to the C terminal of vitronectin, realizes resistance to complement-mediated killing (Riley et al., 2014; Fish et al., 2017). Besides, some surface proteins (such as Sca5 and Adr2) are identified to be important protective antigens and have the potential ability to stimulate B cells to produce a specific antibody that recognizes *R*. *rickettsii*. INF-γ and TNF-α levels are significantly elevated in mice immunized with a combination of Sca5 and Adr2 compared with the control group. Therefore, the surface proteins are potential candidates for subunit vaccines (Gong et al., 2015).

### Effectors involved in subversion of host cells’ movement

In addition to the harnessing of Sca proteins and Adr1-2 to accomplish effective adherence to and invasion into host cells, diverse intracellular pathogens subvert the host actin polymerization machinery to drive movement within and between cells during infection (Pantaloni et al., 2001). The intracellular and intercellular mobility is responsible for infection from cytoplasmic bacteria to neighboring cells, which helps pathogens escape immune surveillance. Compared with most of the TGs, bacteria of the SFG have the capacity to move intracellularly and intercellularly (Teysseire et al., 1992), and other intracellular bacteria, such as *Listeria* and *Shigella*, have been demonstrated to manipulate the effectors ActA (Welch et al., 1998) and VirG/IcsA (Mauricio et al., 2017) to mimic or recruit host nucleation-promoting factors (NPFs).

The actin cytoskeleton is a dynamic filament network that is essential for cell movement, polarization, morphogenesis, and cell division. The assembly of actin filaments at the leading edge drives the movement of eukaryotic cells. Actin polymerization also contributes to providing force to internalize endocytic vesicles from the cell membrane (Pollard and Cooper, 2009). The members of the Rho family of small GTP-binding proteins, including Rac, Rho, and Cdc42, regulate actin-dependent processes (Rajat et al., 1999). WASP family proteins, especially WASP (Wiskott-Aldrich syndrome protein, which is only expressed in hematopoietic cells) and N-WASP (ubiquitously expressed, shared about 50% homology with WASP), mediate the effects of Cdc42 on the actin cytoskeleton. Active NPFs, such as N-WASp and Scar (Machesky et al., 1999), stimulate the nucleation activity of Arp2/3 (actin-related protein 2/3) complex through WCA domains which bind the Arp2/3 complex to initiate a new actin filament as a branch on an existing filament (Fassler et al., 2020). Because of sufficient ATP-actin-profilin, new branches extend rapidly, push the membrane forward, and form protrusions.

RickA, a 517-amino acid protein, is expressed on the surface of *R*. *conorii*. Actin polymerization assays *in vitro* show that RickA alone is unable to stimulate actin polymerization. However, it is efficient in accomplishing actin assembly when combined with the Arp2/3 complex (Gouin et al., 2004). The decrease in actin tails recruited by *R*. *conorii* in ScarWA-expressing cells is due to the overexpression of ScarWA sequestering Arp2/3, which further indicates that the Arp2/3 complex is hijacked during infection, as it is in *Listeria*-infected cells (Boujemaa-Paterski et al., 2001). Ectopic expression of RickA in Vero cells displays thin filopodia that are absent from non-transfected cells, reinforcing that RickA is a novel type of activator of actin nucleation through the activation of the Arp2/3 complex (Gouin et al., 2004). Western blotting analysis using a specific monoclonal antibody has shown that RickA is expressed in all tested SFG rickettsiae (including *R*. *rickettsii*, *R*. *conorii*, *R*. *africae*, *R*. *sibirica*, *R*. *slovaca*, etc.) but not in *R*. *prowazekii* and *R*. *typhi* (Balraj et al., 2008), which may be a partial reason why the TG bacteria do not exhibit actin-based motility. *R*. *rickettsii*-induced long actin-tail and actin-based motility are dependent on RickA, which activates both the nucleation and Y-branching activities of the Arp2/3 complex (Jeng et al., 2004). The universal existence of RickA and the similar function among SFG are coincident with the phenomenon of actin-based mobility (Teysseire et al., 1992), underlying the molecular mechanisms involved in cell-to-cell spread among the SFG.

Unlike the Arp2/3 complex, formin proteins, which are extensively present in all eukaryotes, produce unbranched actin filaments. The conserved formin homology (FH) domains, FH1 and FH2, are sufficient to *de novo* nucleate actin monomers to actin filament formation (Campellone and Welch, 2010). In *R*. *parkeri*, a representative SFG species, the Sca2 passenger domain contains a central cluster of three putative WH2 motifs flanked by two proline-rich domains (PRDs) that are similar to the FH1 domain of formins. The WH2 domain, especially the FH1 domain of Sca2, is highly conserved among SFG rickettsiae but absent from the TG, which is consistent with the lack of actin-based motility in the species. The Sca2 passenger domain mimics the function of formins and displays dose-dependent nucleation activity independently of host nucleators, eliminating the lag phase of polymerization. The intrinsic nucleation activity of Sca2 distinguishes it from RickA, which is dependent on the Arp2/3 complex (Haglund et al., 2010). In *R. rickettsii*, truncated Sca2, through transposon insertion, diminishes the unbranched actin comet tail induced by bacteria, leading to defective actin-based motility, while it does not affect the replication in Vero cells. Consequently, the Sca2 mutant *R. rickettsii* does not elicit the fever symptom in guinea pigs, which are used as a model system to assess rickettsial pathogenesis and display the small-plaque phenotype, showing that Sca2 is a crucial virulence determinant *in vivo* and *in vitro* (Kleba et al., 2010). Interestingly, *R*. *peacockii*, (members of SFG) which encodes an intact Sca2 and disrupts RickA, has a lack of actin-based motility (Simser et al., 2005). This observation indicates that at least these two key factors, Sca2 and RickA, coordinate with cell-to-cell spread. Taken together, these data suggest that Sca2 not only functions in the initial steps of rickettsial infection and the invasion of host cells (Cardwell and Martinez, 2012) but also drives bacterial intracellular and intercellular mobility, which effectively accelerates the cell-to-cell spread and evades the immune response.

Tension at the adjacent junction is considered a barrier in preventing cell-to-cell spread. Previous studies have justified that actin-driven motility is sufficient for bacterial spread (Monack and Theriot, 2001). *R*. *parkeri* employs distinct mechanisms compared with other pathogens. Sca4, previously named gene D, is an intracytoplasmic protein, and its matured molecular protein weight is 120 kDa. Moreover, Sca4 has a passenger domain similar to those of OmpA and OmpB but does not have a C-terminal AT domain (Blanc et al., 2005). Although it lacks the AT domain, Sca4 is secreted into the host cytoplasm dependent on C-terminal domains and is endowed with pivotal functions for cell-cell dissemination. The presence of two VBSs (vinculin binding sites) in Sca4 is necessary to bind the cell adhesion protein, vinculin, and it is demonstrated by the Co-IP assay that mutant Sca4 in the putative binding site (L415A, S416E, Y814I, and V820E) disrupts vinculin binding and decreases the number of protrusion engulfed formations (Park et al., 2011). Actually, this binding disrupts vinculin recruited by α-catenin, where it participates in actin-mediated reciprocal pulling forces to stabilize junctions (Buckley et al., 2014). This molecular mechanism underlies how *R*. *parkeri* relieves tension at the cell-to-cell junction and makes it easy to form protrusion engulfment, eventually promoting intracellular infection (Lamason et al., 2016).

Nowadays, a new discovery has been reported that roaM (regulator of actin-based motility) is increased by *R*. *rickettsii* to negatively modulate actin-based motility which is important for bacteria to propel themselves from the cytoplasm to adjacent cells. The study utilizes gene manipulation to deplete the ectopic expression of *roaM* gene in order to assess the role of roaM in actin-based motility. The data show that increased expression of roaM results in a dramatic reduction of actin comet tails, and depletion of this gene can rescue this phenotype. The *roaM* gene is intact in SFG *Rickettsia* spp., and it is not secreted into the host cell cytoplasm. Consequently, the mechanism of how the *roaM* gene regulates actin-based motility is independent of the activation of Arp2/3 or other NPFs (Nock et al., 2022). However, the expression of *roaM* also does not affect the expressions of Sca2, Sca4, and RickA, indicating that there are unknown effectors involved in actin-based motility.

### Effectors involved in the escape from lysosome-mediated degradation

In the life cycle of rickettsiae, the initial step is to invade host cells through the Scas and Adr interactions with receptors on the surface of host cells, cascading the upstream signaling pathway to rearrange the cytoskeleton. The next challenge internalized bacteria are faced with is host lysosome-mediated degradation.

Bacteria evolved virulence factors to escape from the phagocytic vacuole into the cytoplasm and utilize the host energy substrate for plentiful multiplication. The pretreatment of *R*. *rickettsii* with PLA_2_ (phospholipase A) inhibitors significantly decreases the percentage of infected Vero cells, the size of formative plagues (Silverman et al., 1992), and cell injury (Walker et al., 2001a). With the completion of the sequencing of *R*. *conorii*, the gene *RC127* has been reported to encode phospholipase D (PLD, molecular weight 24 kDa), which possesses a conserved H-K-D motif. The H-K-D motif is conserved in all members of the PLD superfamily and is critical for biochemical activity. The capacity of PLD is demonstrated by the release [^3^H]-choline from phosphatidyl [^3^H]-choline, which detects water-soluble radioactivity and comigrates with the choline standard through a thin layer chromatography assay. It is the consensus that phosphatidylcholine is the dominant constituent of cell membranes. A blockade of PLD by polyclonal antibody reduces the cytotoxicity of *R*. *conorii* and *R*. *prowazekii* when infecting Vero cells (Renesto et al., 2003). Pat1, which is conserved in sequenced rickettsial genomes, has G (glycine-rich motif), S (serine hydrolase motif), and D (active site aspartic acid) conserved catalytic sites. The mutants in S/D catalytic sites can disrupt the escape from phagosomal vacuoles into the host cell cytoplasm (Rahman et al., 2013).

### Effectors involved in the disruption of immune surveillance

Besides elusion from lysosome-mediated degradation, rickettsial species also disrupt the biological function of the Golgi apparatus in host cells. Genomic comparisons between the virulent strain, *R*. *rickettsii* Sheila Smith, and the avirulent strain, Iowa, reveal that rickettsial ankyrin repeat protein 2 (RARP-2) in Iowa is truncated, and the deletion of 7 of 10 ankyrin repeat domains in the C terminal of the protein contributes to proteolytic degradation of the truncated RARP-2. The full-length protein is accurately colocalized with the endoplasmic reticulum (ER) (Lehman et al., 2018). Overexpression of the Sheila Smith RARP-2 in *R*. *rickettsii* Iowa converts this avirulent strain’s typically nonlytic or opaque plaque type to a lytic plaque phenotype, which is similar to that of the virulent Sheila Smith strain. However, restoring the virulence of the Iowa strain in a guinea pig model is not observed, which is likely attributed to the multifactorial nature of rickettsial virulence (Lehman et al., 2018). RARP2 is a predicted cysteine protease related to eukaryotic caspases, and the mutant of a predicted active site cysteine to alanine (C109A) reverses the effect on the plaque phenotype. In the early stage of infection (4 hours after infection), fragmental trans-Golgi apparatus is induced by the *R*. *rickettsii* Sheila Smith strain at a very low load, while the phenotype is absent from the avirulent Iowa strain. The expression of RARP2 of *R*. *rickettsii* Sheila Smith in the avirulent Iowa strain causes dispersal of the *trans*-Golgi network, which is dependent on proteolytic activity and ankyrin repeat domains, indicating that colocalization with ER is significantly important for the function of RARP-2 (Aistleitner et al., 2020). Disrupting the *trans*-Golgi network leads to a defect in the glycosylation pattern of two cellular proteins: the *trans*-Golgi protein (TGN46) and MHC-I, meanwhile, the defect occurs in protein trafficking from the Golgi apparatus to the plasma membrane. MHC-I is an important antigen presentation protein that is a target of viruses and bacteria to hamper the cell delivery of antigens to immune cells and eventually evade immune surveillance (Walker et al., 2001b; Ambagala et al., 2005; Antoniou and Powis, 2008; Sahni et al., 2013). MHC-І-dependent antigen presentation is the initial step for adaptive immune responses. When the MHC-I gene is knocked out in the mouse model, the susceptibility of MHC-I KO mice is elevated 50,000 fold compared with wild-type C57BL/6 mice during SFG rickettsial infection (Walker et al., 2001b). An intracellular bacterium, *Orientia tsutsugamushi*, decreases the transcriptional level of MHC-I by reducing the cytosol level of NLRC5, the transactivator of MHC-І, in order to hamper biosynthesis of MHC-I (Rodino et al., 2019). Although the direct substrate of RARP-2 is unknown, ER location is a clue for discovering the substrate and has opportunities to elucidate that the inhibited delivery of MHC-I is either associated with the fragmental *trans*-Golgi or the RAPR-2 cleaved substrate. The secretion of PARP-2 is through Rvh T4SS (Rickettsiales vir homolog type IV secretion system, comprising VirB1-11 and T4SS coupling protein VirD4) (Gillespie et al., 2016), which is homologous with *Agrobacterium tumefaciens* virB/virD T4SS but devoid of VirB5 and VirB4. VirB6 and VirB8-9 are duplicated (Gillespie et al., 2009b; Gillespie et al., 2010). It is reported that the C terminus of T4SS substrates is recruited to RvhD4 (Rickettsia homology VirD4), which has the functions of substrate recognition and ATP hydrolysis (Gillespie et al., 2016), and the interaction is crucial for substrates to be transferred into the secretion machine (Atmakuri et al., 2003; Vergunst et al., 2005). However, experimental data are required to demonstrate whether the C terminus of PARP-2 is necessary to be transferred by T4SS.

## Effectors specific for *Rickettsia* spp. of TG

### Effectors involved in hemolysis and escape from phagocytic vacuoles

*R*. *typhi* and *R*. *prowazekii* are major pathogenic species of the TG, which are the causative agents of endemic typhus and epidemic typhus, respectively. Despite the similarity in attachment, entry, and cytoplasmic proliferation, the members of the TG and SFG rickettsiae differ in several features. *R*. *typhi* and *R*. *prowazekii* adhere to and lyse erythrocytes, while the members of SFG rickettsiae are incapable of hemolysis. It is identified that the *tlyC* gene is specifically encoded by TG rickettsiae (*R*. *typhi* and *R*. *prowazekii*) but not SFG rickettsiae (*R*. *rickettsii*, *R*. *australis*, and *R*. *akari*), and transferring the *tlyC* gene into the non-hemolytic mutant *P*. *mirabilis* WPM111 (HpmA^-^) confers the mutant’s hemolytic phenotype (Radulovic et al., 1999).

PLA2 is one of the most intensively studied membrane proteins, which hydrolyzes phospholipids at the sn-2 position to form fatty acids and lysophospholipid products (Quach et al., 2014). PLA2 binds to a membrane surface in a scooting mode and catalyzes the hydrolysis reaction of the sn-2 ester bond of phospholipids, which subsequently results in the destruction of the membrane surface. *R*. *prowazekii* encodes the RP534 protein, a homolog of the *Pseudomonas aeruginosa* ExoU, which is a secreted cytotoxin and has PLA activity (Gendrin et al., 2012; Tyson et al., 2015). The addition of bovine liver superoxide dismutase 1 (SOD1), an activator of ExoU, can shrink the duration to max value and increase the rate of RP534-mediated PC (phosphocholine) hydrolysis (Housley et al., 2011).

In *R*. *typhi*, *RT0590* and *RT0522* encode the Pat1 and Pat2 proteins, respectively. Pat1 homologs are encoded in all sequenced rickettsial genomes, and Pat2 is absent in SFG rickettsiae and highly conserved in TG rickettsiae, indicating a distinct life cycle within eukaryotic cells. The Pat2 homolog of *R*. *prowazekii* (*RP534*) also possesses PLA2 activity, which can be abolished through site-directed mutations in the catalytic Ser/Asp residues. Pat1 and Pat2 are secreted into the cytoplasm and are blocked by antibodies that can significantly decrease the numbers of plague formation and bacteria per cell and affect the escape from the phagocytic vacuole into cell cytosol (Rahman et al., 2013). In the comparison of activated time points of PLD, TlyA, TlyC, and Pat1 during the infection of rickettsia, reverse transcription PCR analyses found that *tlyC* and *pld* were transcribed during the period of phagosome escape, while *tlyA* and *pat1* were not. As we know, the strategies employed by *Salmonella* species in invading host cells are different from rickettsial species. Instead of escaping from phagosomal vacuoles, they adapt by replicating in phagosomal vacuoles and decorating them as SCV (*Salmonella*-containing vacuoles), which can not be recognized by lysosomes. The expression of TlyC or PLD endows *Salmonella* with the capacity to escape from phagosomal vacuoles into the cytoplasm, which further demonstrates the pivotal role of TlyC and PLD in the life cycle of *R*. *prowazekii* (Whitworth et al., 2005).

Members of TG rickettsiae employ abundant membranolytic effectors (TlyA, TlyC, Pld, Pat1, and Pat2) as a PL arsenal to lyse red blood cells and phagocytic vacuoles. The escaped bacteria rapidly replicate in the cytoplasm and accumulate plentiful numbers of offspring, depleting the mass nutrient substrate of host cells and eventually lysing cells for a new start of the life cycle (Ray et al., 2009). However, certain effectors involved in the process of lysis of the host cells remain elusive.

### Effectors modulated phospholipid metabolism to promote bacterial invasion

Arfs (ADP ribosylation factors) are subfamilies of small GTP-binding proteins with low molecular weight and can bind to guanine nucleotides involved in vital intracellular processes, including signal transduction (Liu et al., 2017) and vesicle trafficking (Li and Guo, 2022). Arfs are classified into three types: type I (Arf1-3), type II (Arf4-5), and type III (Arf6). The type is dependent on the spatial distribution of the Arfs and a series of regulators (GEF: guanine nucleotide exchange factors, GAP: GTPase-activating proteins) to switch the Arfs from their GDP-bound inactivated form to a GTP-bound activated form (Donaldson and Jackson, 2011). Because of the significant function of Arf-GEF, bacterial pathogens mimic Arf-GEF to disturb the biological processes of the host cell (Orchard and Alto, 2012). The conserved Sec7 domain (Wright et al., 2014) helped scientists find that *L*. *pneumophila* and *R*. *prowazekii* encode the RaIF protein with a homology of the Sec7 domain. The RalF protein from *L*. *pneumophila*, a substrate of the Dot/Icm secretion system, is necessary for bacteria to hijack Arf1 localized on the surface of LCV (*Legionella*-containing vacuole) and subvert the host cell vesicle traffic from ER to LCV (Nagai et al., 2002).

However, the RaIF protein from *R*. *typhi* is distinguished from *L*. *pneumophila*, which interacts with RvhD4, a substrate of the type IV secretion system (Rennoll-Bankert et al., 2015). It colocalizes with Arf6 and PI5P4K (PIP4K) at the entry foci on the host PM. The accumulation of PI(4,5)P_2_ by PIP4K is crucial for recruiting actin remodeling proteins, like Cdc42. Considering that the *RaIF* gene is pseudogenized or absent from SFG rickettsiae, it is a novel way for TG rickettsia to modulate host cell PI metabolism to benefit from bacterial invasion (Rennoll-Bankert et al., 2016).

### Effectors modulated phospholipid metabolism to induce autophagy

Risk1 (Voss et al., 2020) is an effector with dual-class I and class III PI3K activities (Fox et al., 2020) secreted by rickettsia T4SS. Risk1 catalyzes PI(4,5)P_2_ to PI(3,4,5)P_3_ at the bacterial entry foci, contributing to *R*. *typhi* invading host cells at the primary stage. An antibody blockade of Risk1 on the surface of *R*. *typhi* significantly decreases the efficiency of bacterial entry into host cells. Risk1 depends on class III PI3K activity interacting with Beclin-1 and induces autophagy through simulating the function of class III PI3K activity (PtdIns3KC3, Vps34 in yeast) at the phagophore membrane nucleation step (Huang and Brumell, 2014). *R*. *typhi*-induced autophagy facilitates bacterial cytosol replication (Voss et al., 2020). There is extensive evidence that the PI signaling pathway is important in cell proliferation (Shaw and Cantley, 2006; Schink et al., 2016; Hasegawa et al., 2017), so the secretion of phospholipid kinases or phosphatases is also a universal tactic for bacteria to modulate the phospholipid metabolism of host cells. For example, *L*. *pneumophila* utilizes the MavQ-LepB_NTD (1–311 amino acid in LepB)-SidF axis to generate PI4P on the surface of LCV (Luo and Isberg, 2004; Dong et al., 2016; Hsieh et al., 2021). PI4P is a marker of the Golgi organelle in the secretory pathway, and it anchors secreted effectors from Dot/Icm apparatus on LCV (Weber et al., 2006). *Shigella flexneri* exploits IpgD, a substrate of T3SS (type III secretion system) (Personnic et al., 2016), which is a phosphatase that catalyzes PI(4,5)P_2_ to PI5P (Niebuhr et al., 2002) and up-regulates the PI3K-AKT signaling pathway (Pendaries et al., 2006) to inhibit apoptosis of infected cells. Accumulated PI5P also helps internalize ICAM-1, which is responsible for neutrophil recruitment to infected intestinal epithelial cells (Boal et al., 2016).

## Discussion

**Figure 1:**
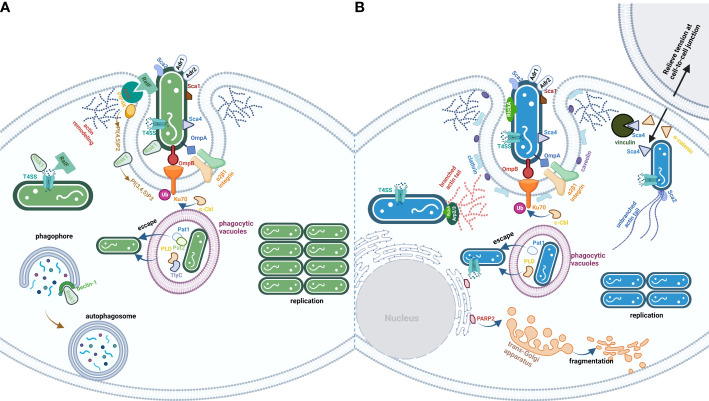
The life cycle of TG **(A)** and SFG **(B)** rickettsiae. The effectors are exploited by *rickettsial* species from TG or SFG. **(A)** is the process of TG invasion of host cells by *rickettsial* species. Besides Adr1 subversion of complement-mediated killing in serum and a series of Scas and Adr2, bacteria from TG employed multiple PLs (Pat1/2, PLD, and TlyC), kinase (Risk1), and RaIF to disturb host cells PI metabolism. **(B)** is the process of SFG invasion of the host cells by *rickettsial* species. The significant characteristic of bacteria from SFG is the ability to spread before infected cell lysis, which utilizes a series of effectors (RickA, Sca2, and Sca4) to modulate cytoskeletal actin polymerization and PM rearrangement.

The obligate intracellular life cycle of *Rickettsia* spp. includes entry into the host cells by phagocytosis (or induced phagocytosis for non-phagocytic cell types), rapid escape from phagocytic vacuoles into the host cytoplasm to evade phagosome-lysosome fusion, which promotes rickettsial survival and replication within the host cytoplasm, exit from the host cells by actin-mediated motility (SFG rickettsiae) or lysis of host cells (TG rickettsiae), and the evasion of complement-mediated killing into next infection stage (Sahni et al., 2019) (**Figure 1**). In this review, we summarized the reported effectors of divergent rickettsial species from SFG and TG and focused on specific effectors (**Table 1**). The review elucidates that the SFG rickettsiae utilize RickA, Sca2, Sca4, and regulator *roaM* to manipulate actin-based intercellular transmission before infected cell lysis, which is significantly distinguished from the TG rickettsiae that spread to the uninfected cells after lysis of the infected cells (McGinn and Lamason, 2021). Well-developed PLs from the TG rickettsiae help bacterial hemolysis, and such a capacity is absent in the SFG rickettsiae. A modulated PI metabolism through T4SS effector Risk1 is a new discovery in TG rickettsiae. We hope to provide new insights through horizontal and vertical comparisons of the divergent effector proteins between SFG and TG. rickettsiae. However, most of the cited studies are exploratory rather than mechanistic, which is confounded by the problem of interaction between lineage-specific *Rickettsia* spp. and has often been studied without considering the complex host-microbial interaction environment that occurs *in vivo*. The characteristic of replicating and proliferating only within the cytoplasm or nucleus of the host cells poses a superior challenge for gene manipulation in rickettsiae (Wood and Azad, 2000). Therefore, the lack of effective gene manipulation systems for rickettsiae prevents us from obtaining direct evidence of the exact function of various genes at the different stages of infection.

**Table 1:** The virulence factors of rickettsial species.

NO.	Effector	Representative Species	Gene ID	Function	Interactor/substrate	Reference
1	Sca5	*R. rickettsii* *R. conorii* Malish7	*A1G_06030* *Rc1085*	Bacterial adherence and invasion	Ku70	(Martinez et al., 2005; Uchiyama et al., 2006; Chan et al., 2009)
2	Sca0	*R. rickettsii* *R. conorii Malish7*	*A1G_06990* *Rc1273*	Bacterial adherence and invasion	α2β1 integrin	(Hillman et al., 2013);
3	Sca1	*R. japonica*	*RJP_0015*	Bacterial adherence	Unknown	(Ngwamidiba et al., 2006; Riley et al., 2010)
4	Sca2	*R. japonica*	*RJP_0085*	Bacterial adherence, invasion, and dissemination	G-actin	(Cardwell and Martinez, 2009)
5	Sca3	*R. typhi*	*RT0438*	Bacterial adherence and invasion	Unknown	(Gillespie et al., 2015)
6	Sca4	*R. parkeri*	*MC1_03740*	Relieves tension at the cell-to-cell junction	Vinculin	(Park et al., 2011; Lamason et al., 2016)
7	Adr1	*R. conorii*	*RC1281*	Evasion of host complement-mediate killing	Vitronectin	(Fish et al., 2017)
8	Adr2	*R. prowazekii*	*RP828*	Bacterial adherence and invasion	Unknown	(Vellaiswamy et al., 2011)
9	RickA	*R. rickettsii*	*RRR_04680*	Mimic WASPs activating Arp2/3 complex	Arp2/3 complex	(Gouin et al., 2004)
10	Pat1	*R. typhi* *R. prowazekii*	*RT0590* *RP602*	phospholipase A2 activity	PC (phosphocholine)	(Walker et al., 2001a; Rahman et al., 2013)
11	Pat2	*R. typhi* *R. prowazekii*	*RT0522* *RP534*	phospholipase A2 activity	PC (phosphocholine)	(Walker et al., 2001a; Rahman et al., 2013)
12	PLD	*R. conorii*	*Rc127*	phospholipase D activity	PC (phosphocholine)	(Renesto et al., 2003)
13	tlyA	*R. prowazekii*	*RP555*	lysis erythrocytes	PC (phosphocholine)	(Radulovic et al., 1999) (Whitworth et al., 2005)
14	tlyC	*R. prowazekii*	*RP740*	lysis erythrocytes	PC (phosphocholine)	
15	RARP-2	*R. rickettsii*	*A1G_05165*	Fragmentation of the *trans*-Golgi network	Unknown	(Lehman et al., 2018; Aistleitner et al., 2020)
16	RalF	*R. typhi*	*RT0362*	GEF of Arf6	Arf6	(Rennoll-Bankert et al., 2015)
17	Risk1	*R. typhi*	*RT0135*	class I and class III PI3Kinase	Rab5 and Beclin1	(Voss et al., 2020)

## Author contributions

DH drafted the original draft. JL edited the manuscript. XO and LS supervised and revised the manuscript. All authors contributed to the article and approved the submitted version.

## Funding

This manuscript was funded by SKLPBS2132 (State Key Laboratory of Pathogen and Biosecurity, Academy of Military Medical Science) and 2019YFC1200602 (National Key Research and Development Program of China).

## Conflict of interest

The authors declare that the research was conducted in the absence of any commercial or financial relationships that could be construed as a potential conflict of interest.

The reviewer YQ declared a past co-authorship with the author XO to the handling editor.

## Publisher’s note

All claims expressed in this article are solely those of the authors and do not necessarily represent those of their affiliated organizations, or those of the publisher, the editors and the reviewers. Any product that may be evaluated in this article, or claim that may be made by its manufacturer, is not guaranteed or endorsed by the publisher.
